# Association of cognitive function with Neurofilament light chain in the aqueous humor of human eye

**DOI:** 10.3389/fnagi.2022.1027705

**Published:** 2022-11-03

**Authors:** Jianhao Bai, Zhongqi Wan, Minli Wang, Xue Wu, Tianyu Wang, Yuanyuan Zhang, Yawen Xue, Hong Xu, Qing Peng

**Affiliations:** ^1^Department of Ophthalmology, Shanghai Tenth People's Hospital of Tongji University, Tongji University School of Medicine, Shanghai, China; ^2^School of Biomedical Engineering, Shanghai Jiao Tong University, Shanghai, China

**Keywords:** age-related macular degeneration, cataract, Alzheimer’s disease, aqueous humor, neurofilament light chain

## Abstract

**Objectives:**

To evaluate the predictive clinical role of neurofilament light chain (NfL), amyloid-β (Aβ), glial fibrillary acidic protein (GFAP), and phosphorylated tau at threonine 181 (p-tau181) proteins in human aqueous humor (AH) and quantify the retinal macular microvascular parameters by optical coherence tomography angiography (OCTA) as early diagnostic markers of Alzheimer’s disease (AD).

**Methods:**

This prospective, single-site, cross-sectional, cohort study enrolled 55 participants, including 38 patients with neovascular age-related macular degeneration (nAMD) and 17 individuals with senile cataracts. The single-molecule array platform was used to quantitatively measure the levels of AH NfL, Aβ40, Aβ42, GFAP, and p-tau181 proteins in AH. The mini-mental state examination (MMSE) score was used to assess the global cognitive function. OCTA scan with 6 × 6 mm macular area was used to quantify the retinal thickness and microvascular densities of superficial retinal capillary plexuses and deep retinal capillary plexuses.

**Results:**

NfL, Aβ40, Aβ42, GFAP, and p-tau181 were detected in all AH samples by Simoa platform. Individuals with cataract had higher concentrations of NfL and p-tau181 but lower Aβ40 and Aβ42 and similar GFAP compared to those with nAMD. Lower MMSE scores showed a negative correlation with NfL concentration of AH not only in the nAMD group (*p* = 0.043), but also in the cataract group (*p* = 0.032). However, the MMSE scores were not associated with the levels of Aβ40, Aβ42, GFAP, or p-Tau181. Further analysis found that the Aβ40 and Aβ42 concentrations showed a strong positive correlation (*p* < 0.0001). In addition, the NfL concentration showed a mild positive correlation with that of GFAP in the cataract group (*p* = 0.021). Although it has not reached statistical significance, there was a correlation between the levels of NfL and Aβ42 in the nAMD group (*p* = 0.051). Moreover, the macular superficial vessel density values had a negative correlation with the concentration of NfL (*p* = 0.004) but a positive correlation with MMSE scores (*p* = 0.045). The macular deep vessel density values were negatively correlated with the concentration of p-tau181 (*p* = 0.031) and positively correlated with MMSE scores (*p* = 0.020).

**Conclusion:**

The examination of AD-related biomarkers in human AH and OCTA may improve the ocular-based AD detection methods and contribute to forestalling the progression of preclinical AD.

## Introduction

In the past few decades, the increase in life expectancy and the growing aging population have increased the global prevalence of neurodegenerative diseases, including Alzheimer’s disease (AD) and Parkinson’s disease (PD; Hou et al., 2019). Globally, >35 million people are estimated to be suffering from dementia, and the number is expected to rise to 131.5 million by 2050 (Reilly et al., 2015). Accounting for 60–70% of dementia cases worldwide, AD is the most common type, and the prevalence is estimated to rise to 13.8 million by 2060 (Alzheimer’s disease facts and figures, 2022). The etiology of AD is multifactorial, in addition to genetic factors, the risk of developing AD can also be attributed to acquired factors including cerebrovascular diseases, hypertension, diabetes, dyslipidemia, diet and nutrition(Silva et al., 2019; Hou et al., 2022). Since only a few treatments are effective, these neurodegenerative diseases often develop in an irreversible manner.

Therefore, early diagnosis of AD is essential. However, the disease can be diagnosed definitely only by histopathological examination (Javaid et al., 2016). The clinicians make the diagnosis based on the medical history, observing the clinical manifestations, and using expensive imaging or invasive tests, such as magnetic resonance imaging (MRI), positron emission tomography (PET), and evaluation of cerebrospinal fluid (CSF) biomarkers (O'Bryhim et al., 2018). Although assessed by experienced doctors, 10–15% of cases with prenatal diagnosis tend to be inaccurate (Thal et al., 2006). Due to the difficulties and delay in clinical diagnosis, patients often have irreversible pathological damage before starting the treatment. The classic symptoms of decreased memory, learning, calculation, and thinking are apparent only in the late stages of AD when an irreversible neuronal loss has already occurred (Salobrar-García et al., 2019). Thus, early detection is essential to prevent the disease progression. However, finding reliable, low-cost, and widely available screening methods remains a challenge (Blennow and Zetterberg, 2018).

Interestingly, AD is a heterogeneous disease with various cognitive subtypes. Visual symptoms are prominent due to local pathology of the parietal-occipital region in the visual variant AD (VVAD; Kaeser et al., 2015). The eye is a transparent medium to the brain. In anatomy and development, the retina is considered a part of the central nervous system (CNS). The retina and the internal visual membrane of the eye are derived from a neural tube and share a common neuro-ectodermal embryological origin and vasculature with the brain and transfer the visual stimuli to the brain (Sernagor et al., 2001; Javaid et al., 2016). The vital connection between the eyes and the brain suggests that the eyes could be regarded as an extension of the CNS (Marchesi et al., 2021), and the early manifestations of neurodegenerative diseases could be identified through the eyes. In addition, the immune response of the eye is similar to that of the brain and spinal cord, although it has unique anatomical structures and a local array of surface molecules and cytokines. Furthermore, various eye-specific pathologies are similar to the other CNS pathologies. These anatomical similarities permit common manifestations of the disease among the eye and brain, and the eye provides a direct window to neuroretinal diseases (London et al., 2013). Several neurodegenerative diseases, such as AD and PD, have manifestations in the eye, and eye symptoms usually precede the clinical symptoms and traditional diagnosis of such disorders (Javaid et al., 2016; Levin et al., 2016; Colligris et al., 2018; Guo et al., 2021). Accumulating evidence of the genetic, epidemiological, molecular, and clinical links between AD and various disorders of the eye, such as cataracts, neovascular age-related macular degeneration (nAMD), glaucoma, and diabetic retinopathy, indicate that these eye diseases and AD might share common risk factors and pathological mechanisms at the molecular level (Hersi et al., 2017; Zhang et al., 2017; Mancino et al., 2018; Lee et al., 2019). For example, some researches demonstrated that individuals with these eye diseases are at an increased risk of AD (Lee et al., 2019). These studies suggest that eye examination could serve as a non-invasive method for the early diagnosis of AD.

Previous studies on the early detection of AD have been focused on the diagnostic utility of protein markers in the CSF and blood, and levels of NfL, amyloid-β (Aβ), and total tau (T-tau) are correlated with the cognitive function in individuals with AD (Agarwal and Tripathi, 2011; Haapalinna et al., 2018; Hampel et al., 2018). Several recent clinical studies have shown that elevated phosphorylated tau 181 (p-Tau181) contribute to diagnostically relevant and accurate information in the early AD stages (Janelidze et al., 2020). However, previous studies on these AD biomarkers were only focused on CSF or blood of AD patients but rarely reported in aqueous humor (AH). Herein, we postulated that analysis of AH may have a clinical utility in early AD detection. Therefore, the present study aimed to investigate the correlation among AD protein biomarkers including NfL, Aβ40, Aβ42, GFAP, and p-tau181 in human AH, macular microvascular parameters and neurocognition in the nAMD and cataract patients.

## Materials and methods

### Standard protocol approvals

This prospective, single-site, cross-sectional, cohort study was approved by the Research Ethics Committee of Shanghai Tenth People’s Hospital and registered in the China Clinical Trials Registration Center (ClinicalTrials.gov: ChiCTR2100051795). This study conducted in the Department of Ophthalmology of Shanghai Tenth People’s Hospital Affiliated to the School of Medicine of Tongji University, according to the principles of Helsinki Declaration. All patients signed informed consent before enrolling in the study.

### Study design

A total of 55 patients, including 38 with neovascular age-related macular degeneration (nAMD group) and 17 individuals with senile cataract (cataract group), were included in this study. All patients underwent magnetic resonance imaging (MRI) and comprehensive ophthalmic examination, including intraocular pressure examination, visual acuity test, optometry, slit lamp biomicroscope, digital fundus photography, and optical coherence tomography angiography (OCTA). In addition, the mini-mental state examination (MMSE) was performed to evaluate the neurocognitive function and status of the subjects before the operation. Moreover, all nAMD patients were diagnosed by three retinal experts and examined by fundus fluorescein angiography (FFA) and indocyanine green angiography (ICGA).

### Inclusion and exclusion criteria

The inclusion criteria of the nAMD group were as follows: (1) age 55–85 years; (2) newly diagnosed nAMD patients who have not received any anti-vascular endothelial growth factor (VEGF) treatment before; (3) willing to provide AH samples; (4) without cataract or other retinal diseases. The inclusion criteria of the cataract group were as follows: (1) age 55–85 years; (2) senile cataract patients who need phacoemulsification; 3) willing to provide AH samples; 4) patients without any retinal diseases.

The exclusion criteria for the patients were as follows: (1) received any intraocular or photocoagulation therapy; (2) history of pre-existing other ocular diseases, such as glaucoma, uveitis, retinal degeneration, and optic neuropathy; (3) diagnosed with any type of dementia, including AD; (4) systemic immune diseases; (5) history of head injuries or craniocerebral surgery; (6) family history of cognitive disorders or any genetic history; (7) allergic to sodium fluorescein and indocyanine green; (8) those the researchers believe that they should be excluded.

### Sample collection and processing

All patients with nAMD received the intravitreal anti-VEGF drugs injection. Limbal puncture was performed before vitreous injection. An equivalent of 50–100 μl AH was collected through the limbus with an insulin syringe. Subsequently, 0.05 ml of Lucentis (Novartis Pharma Schweiz, AG) was injected into the vitreous cavity to restore the intraocular pressure balance. 17 AH samples of the cataract group were collected from 17 patients with senile cataracts. At the beginning of phacoemulsification, the surgeon used insulin syringes to obtain 50–100 μl of undiluted AH samples through the corneoscleral margin incision that was then transported to the laboratory for analysis.

### Biomarker measurement

All the AH samples were clarified by centrifugation at 1000 rpm, 4°C for 15 min. The concentration of NfL, Aβ 1–40, Aβ 1–42, GFAP, and p-tau181 were measured using kits in each AH sample on the Simoa HD-X platform using a supersensitive Single molecule array (SIMOA) technology (Quantrick, MA, United States), according to the manufacturer’s instructions, by technicians involved in the test blinded to the clinical data. The NF-light assay (Cat. No. 103186), multiplex Neurology 4-Plex A kits (Cat. No. 101995), and p-tau181 assay kit V2 (Cat. No.103714) were purchased from Quanterix.

### OCTA measurements

OCTA was performed using an angioscope with a 6 × 6 mm^2^ area (Optovue RTVue XR Avanti, Optovue, Inc.) to scan the patients’ macular area, and the flow density map software AngioAnalytics in-built version 2016.1.0.26 was used to quantify the retinal thickness and microvascular densities of superficial retinal capillary plexuses and deep retinal capillary plexuses automatically. All measurements are repeated twice with sufficient image quality (signal strength index >7).

### Neurocognitive function test

MMSE test was administered to all subjects within 1 week before collecting AH samples, and the scores were used to assess the global cognitive function of each patient. All the tests were performed by the same well-trained, blinded certified psychometrist. The changes in the neurocognitive function of the subjects were analyzed according to the MMSE score, and the correlation between MMSE and AD-related biomarkers was explored in AH.

### Statistical analysis

Linear regression was used to test the correlations among AD biomarker levels, MMSE scores, macular superficial and deep vessel density values and retinal thickness. The AD biomarker levels >0 were used after log transformation to fit the skewed distributions for regression. The associations of AD biomarker levels with MMSE scores and between AD biomarker levels and the vascular density, retinal thickness of the superficial and deep retina were assessed. *p*-values <0.05 indicated statistical significance.

## Results

**Figure 1:**
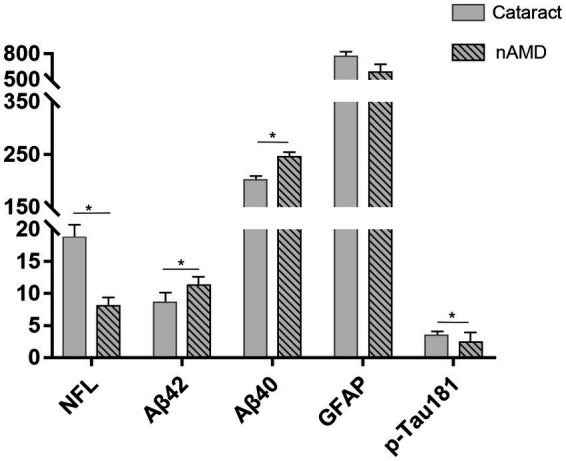
The difference evaluation of AD-related biomarkers in the two groups. Higher concentrations of NfL and p-tau181 but lower concentrations of Aβ40 and Aβ42, while similar concentrations of GFAP in AH of cataract group compared to those in nAMD group. *p < 0.05.

The introduction of supersensitive immunoassay (Simoa) used in this study allows a more accurate quantification of the low NfL and p-tau181 concentrations in AH, even at very low concentrations (down to a few pg./ml). This study determined the concentrations of NfL, Aβ40, and Aβ42, GFAP, and p-tau181 proteins in all AH samples from nAMD and cataract subjects. The summary statistics of AD biomarker levels in AH samples of the two groups are shown in Table 1. Due to the skewed distribution of the original data, the AD-related proteins in the two groups of AH samples showed normal distribution after the values were checked *via* quality control and converted by log_2_ transformation. In addition, about 52.9% of the cataract patients were males with an average age of 68.82 years, and the mean MMSE score was 20.12. Similarly, 47.4% of nAMD patients were males with an average age of 67.11 years, and the mean MMSE score was 27.63. The data showed no structural bias between age, gender, and education level of the two groups, but lower mean MMSE score in the cataract group was observed compared to that in the nAMD group (Table 2). Notably, the difference evaluation of AD biomarkers in the two groups found that individuals with cataracts had higher concentrations of NfL (*p* = 0.039) and p-tau181 (*p* = 0.029) but lower concentrations of Aβ40 (*p* = 0.031) and Aβ42 (*p* = 0.011), while the GFAP (*p* = 0.296) concentrations were similar in the AH compared to those with nAMD (Figure 1).

**Table 1:** The levels of AD-related biomarkers in AH in two groups (pg/ml).

	nAMD group			Cataract group		
	Mean	Median	*SD*	Mean	Median	*SD*
NfL	8.08	3.61	9.89	18.80	8.08	27.37
Aβ40	246.21	240.74	68.85	202.13	191.80	66.71
Aβ42	11.32	10.79	3.60	8.63	8.35	3.25
GFAP	586.38	385.79	481.84	772.91	368.28	822.95
p-tau181	2.38	2.15	1.18	3.47	3.07	1.25

NfL, neurofilament light chain; Aβ, amyloid-β; GFAP, glial fibrillary acidic protein; p-tau181, phosphorylated tau at threonine 181.

**Table 2:** General condition of the nAMD and cataract groups.

	nAMD group (*n* = 38)	Cataract group (*n* = 17)
Age	67.11 ± 8.16	68.82 ± 8.69
Gender (Female/Male)	20/18	8/9
MMSE scores	27.63 ± 1.70	20.12 ± 3.50
Eye (OD/OS)	17/21	9/8
Intraocular pressure	16.17 ± 1.32	15.86 ± 1.74
BCVA (Log MAR)	0.86 ± 0.11	0.88 ± 0.11
Highest level of education	10.29 ± 2.50	10.65 ± 2.15
Cerebrovascular diseases (*n*, %)	9(23.7%)	4(23.5%)
Hypertension (*n,* %)	13(34.2%)	7(41.2%)
Diabetes (*n*, %)	9(23.7%)	5(29.4%)
Dyslipidemia (*n*, %)	6(15.8%)	3(17.6%)

MMSE, The mini-mental state examination; BCVA, Best corrected visual acuity.

**Figure 2:**
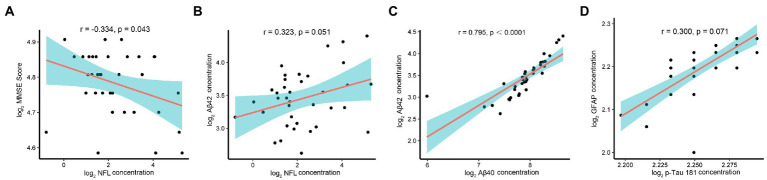
Correlation analysis, AD-related biomarkers and MMSE scores in nAMD group. Note: The AD-related biomarkers concentrations and MMSE scores were log-transformed. p < 0.05 was considered statistically significant. The blue area represented the 95% confidence interval. **(A)** Lower MMSE scores correlated with higher AH levels of NfL (*r* = −0.334, *p* = 0.043). **(B)** Although it has not reached statistical significance, there was a correlation between the levels of NfL and Aβ42 (*r* = 0.323, *p* = 0.051). **(C)** Higher levels of Aβ40 in AH were associated with higher levels of Aβ42 (*r* = 0.795, *p* < 0.0001). **(D)** Although it has not reached statistical significance, there was a positive correlation between the concentrations of p-tau181 and GFAP (*r* = 0.300, *p* = 0.071).

The correlation analysis of the AD biomarkers in the nAMD group showed lower MMSE scores, indicating poorer cognitive function, had a negative correlation with the concentration of NfL (*r* = −0.334, *p* = 0.043) in AH (Figure 2A). However, MMSE scores was not associated with the level of Aβ40, Aβ42, GFAP, or p-Tau181. Further analysis found there was a correlation between the levels of NfL and Aβ42, although it has not reached statistical significance (*r* = 0.323, *p* = 0.051) (Figure 2B). In addition, higher levels of Aβ40 in AH were associated with higher levels of Aβ42 (*r* = 0.795, *p* < 0.0001) (Figure 2C). Although it has not reached statistical significance, there was a positive correlation between the concentrations of p-tau181 and GFAP (*r* = 0.300, *p* = 0.071) (Figure 2D).

**Figure 3:**
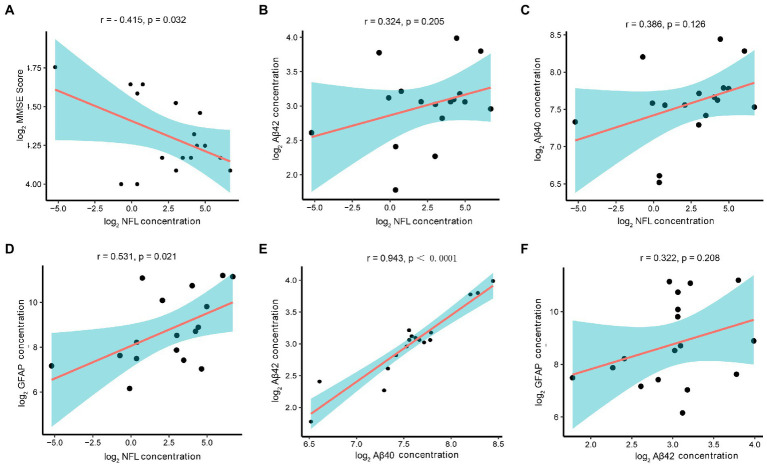
Correlation of AD-related biomarkers concentrations with MMSE scores in the cataract group. Note: The AD-related biomarkers concentrations and MMSE scores were log-transformed. *p* < 0.05 was considered statistically significant. The blue area represented the 95% confidence interval. **(A)** Lower MMSE scores correlated with higher AH levels of NfL(*r* = −0.415, *p* = 0.032). **(B–D)** The NfL concentration showed a mild positively correlation with the concentrations of Aβ42 (*r* = 0.324, *p* = 0.205), Aβ40 (*r* = 0.386, *p* = 0.126), and GFAP (*r* = 0.531, *p* = 0.021). **(E)** The Aβ40 and Aβ42 concentrations showed a strong positive correlation (*r* = 0.943, *p* < 0.0001). **(F)** Although it has not reached statistical significance, there was a correlation between the concentrations of Aβ42 and GFAP (*r* = 0.322, *p* = 0.208).

In the cataract group, the concentration of NfL in AH also showed a negative correlation with MMSE scores (*r* = −0.415, *p* = 0.032) (Figure 3A). In the association analysis among the AD biomarkers, the NfL concentration showed a mild positively correlation with the concentrations of Aβ42 (*r* = 0.324, *p* = 0.205), Aβ40 (*r* = 0.386, *p* = 0.126), and GFAP (*r* = 0.531, *p* = 0.021) (Figures 3B–D). Moreover, the Aβ40 and Aβ42 concentrations showed a strong positive correlation (*r* = 0.943, *p* < 0.0001) (Figure 3E). Interestingly, although it has not reached statistical significance, there was a mild positive correlation between the concentrations of Aβ42 and GFAP (*r* = 0.322, *p* = 0.208) (Figure 3F).

**Figure 4:**
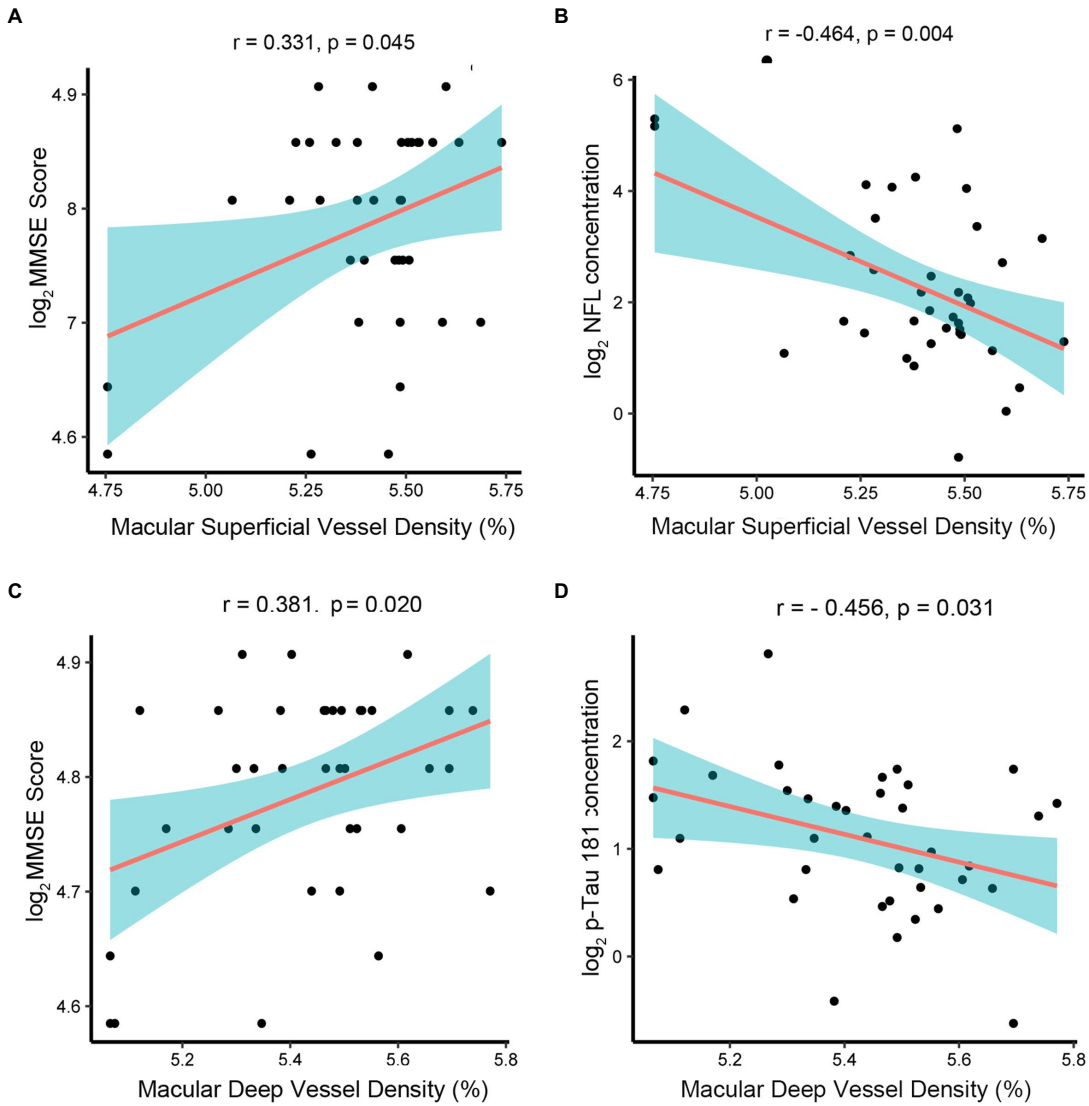
Associations between retinal thickness, retinal vessel density, and AD-related proteins in AMD group. The AD-related biomarkers concentrations and MMSE scores were log-transformed. *p* < 0.05 was considered statistically significant. The blue area represented the 95% confidence interval. **(A,B)** The macular whole enface superficial vessel density values had a positive correlation with MMSE scores (*r* = 0.331, *p* = 0.045) but a negative correlation with the concentration of NfL (*r* = −0.464, *p* = 0.004). **(C,D)** The macular whole enface deep vessel density values were positively correlated with MMSE scores (*r* = 0.381, *p* = 0.020) and negatively correlated with the concentration of p-tau181 (*r* = −0.456, *p* = 0.031).

In order to explore the feasibility and effectiveness of retinal biomarkers measured by OCTA images as a diagnostic tool for early AD, we analyzed the correlation between retinal-specific markers of neurodegeneration obtained by OCTA, including superficial retinal thickness, retinal deep thickness, superficial retinal vessel density, deep retinal vessel density, and AD-related proteins in AH. We found that the macular whole enface superficial vessel density values measured by OCTA had a positive correlation with MMSE scores (*r* = 0.331, *p* = 0.045) (Figure 4A) but a negative correlation with the concentration of NfL (*r* = −0.464, *p* = 0.004) (Figure 4B). The macular whole enface deep vessel density values were positively correlated with MMSE scores (*r* = 0.381, *p* = 0.020) (Figure 4C) and negatively correlated with the concentration of p-tau181 (*r* = −0.456, *p* = 0.031) (Figure 4D). However, the current data showed that superficial retinal thickness was not associated with AD biomarker levels in the AH of nAMD patients or MMSE scores. Additionally, no significant correlation was established between deep retinal thickness and AD biomarker levels or MMSE scores.

## Discussion

The eye is the medium of brain pathology. The eye biomarkers may exist in the early stage of AD and predate the occurrence of cognitive impairment or dementia symptoms observed in the clinic. The detection of eye biomarkers may be valuable to the early diagnosis of AD to minimize the physical damage and expensive assessment caused by invasive examination or multiple imaging scans of the brain. Prior investigations on the role of the eye in AD have focused primarily on retinal markers of neurodegeneration obtained through optical coherence tomography (OCT) scans or OCTA, including choroidal thickness, nerve fiber layer and ganglion cell layer thickness. However, retinal biomarkers measured by OCT imaging alone may not be sufficient as a diagnostic tool in early AD, a approach that involves both protein biomarkers in AH and OCT images might be more comprehensive.

More and more animal experiments and clinical evidence have shown an epidemiological, genetic, molecular, and clinical link between AD, nAMD, and cataracts (Baker et al., 2009; Tsai et al., 2015; Hwang et al., 2021). AD is the most common dementia of the elderly, wherein visual abnormalities occur before cognitive impairment. nAMD is a retinal degenerative disease and a leading cause of irreversible blindness worldwide.AD and nAMD, both being diseases of the elderly, have several epidemiological, etiological and histological overlaps in pathogenesis. The neuroinflammatory response and cell loss were considered of crucial importance in AD, such inflammatory response in the brain can occur in the retina of nAMD patients as the eye represents an extension of the brain. A cataract is a lens disease that is highly related to age. These three diseases have similar pathophysiological mechanisms (Melov et al., 2005; Liu and Zhu, 2017; Ashok et al., 2020). A large number of clinical studies have consistently shown that NfL, Aβ40, Aβ42, and p-tau181 in CSF and plasma are the key factors with predictive value for early AD detection (Agarwal and Tripathi, 2011; Schoonenboom et al., 2012; Olsson et al., 2016; Hampel et al., 2018; Paquet et al., 2018). However, there is still a lack of research on the early diagnosis and treatment of AD by detecting the concentration of AD-related proteins in human AH. AH is a specific biological sample. The total protein content in AH is only 0.2 mg/ml, which is 1/400–1/300 of that in the plasma. Consequently, the expression of AD-related proteins in AH has been rarely reported. Simoa assay has shown 126- fold higher sensitivity than the ELISA(Kuhle et al., 2016), and it facilitates automatic analysis of these biomarkers in AH with high precision and stability.

The current study determined and quantified the concentrations of NfL, Aβ40, Aβ42, GFAP, and p-tau181 proteins in the AH samples from all nAMD and cataract subjects. To the best of our knowledge, this is the first study reported in AH and correlating these AD-related proteins with neurocognition measured by MMSE and macular microvascular parameters in nAMD and age-rerated cataract patients. Notably, except for GFAP, the concentrations of all the AD-related proteins were significantly different in AH samples from nAMD and cataract patients. These proteins are related to cognitive function scores, indicating that AD-related proteins in AH of non-AD patients are related to eye conditions and changes in cognitive function. A recent study (Tsai et al., 2014) detected the vitreous levels of the same proteins in patients with epiretinal membrane, macular hole, diabetic retinopathy, and retinal detachment. However, one of the limitations of that study was the variance in co-existing ocular and systemic morbidities among the participants. However, our results demonstrated that individuals with nAMD had lower concentrations of NfL and p-tau181 but higher concentrations of Aβ40 and Aβ42, and similar GFAP concentrations in AH compared to those with cataracts. A previous study (Zhu et al., 2021) confirmed that the Aβ42 expression was significantly lower in the cataract group compared to the nAMD group, which is consistent with our finding.

It is worth noting that most of the previous clinical studies conducted by testing human AH samples took the AH samples of age-related cataract patients as the control group and AH of other non-cataract patients as the experimental group, because in the real clinical situation, it is very difficult for researchers to obtain AH samples of completely normal subjects (this is not in line with ethical principles). AH samples of senile cataract patients would be considered to be the closest to normal physiological conditions. However, in this study, AH of senile cataract patients cannot serve as the control group, because there might be a potentially important link between cataract and AD as mentioned above. For example, a population-based prospective study of 5,888 adults, aged ≥65 years, found that cataracts were associated with both AD and VAD/mixed dementia, whereas nAMD was associated with AD only (Hwang et al., 2021). Based on our data, it could be speculated that cataracts and nAMD are risk factors for AD, and cataracts may be more likely to develop various types of dementia than patients with nAMD. For verifying this conjecture, data on AD biomarkers in AH from healthy elderly are crucial, but difficult to achieve.

The basic pathological features of AD are an extracellular accumulation of Aβ and the intracellular deposition of p-tau, which in turn leads to neurodegeneration and glial activation. nAMD and cataracts exhibit similar pathological damage. Notably, Aβ protein is not only the main component of senile plaque but also a critical component of drusen. Many studies have shown Aβ deposition in retinal pigment epithelium (Ohno-Matsui, 2011; Guo et al., 2014; Ong et al., 2019). Additionally, the increased level of amyloid in the retina and AH may be involved in the apoptosis of retinal ganglion cells, resulting in AD before early symptoms (Prakasam et al., 2010; More and Vince, 2015). The overexpression and accumulation of Aβ lead to early pathological changes in nAMD (Prasad et al., 2017). Some clinical studies also have detected Aβ in the human lens (Shah et al., 2017; van Wijngaarden et al., 2017). Kerbage et al. (2015) utilized a fluorescent ligand eye scanning (FLES) technique to detect the fluorescence signature specific to an exogenous ligand bound to Aβ in the lens of the eye; the technique could be used to predict AD with 85% sensitivity and 95% specificity [48]. Together, the above studies suggested that the pathological mechanism of AD may be related to the development of age-related cataracts. In this study, Aβ40 and Aβ42 were quantitatively detected in all AH samples of patients with nAMD and cataracts. Thus, we speculated that the Aβ protein in AH samples might be due to the accumulation of Aβ in the retinal pigment epithelium and its entry in the anterior chamber through the scleral venous sinus or vitreous humor. Another possible approach is from the aging lens. Additionally, in our study, the Aβ40 and Aβ42 concentrations showed a strong positive correlation in the AH of both groups, which was consistent with that in CSF and plasma. However, the mechanism of AD-related biomarkers entering AH and protein concentration difference in patients with different ophthalmopathy needs to be further elucidated with animal experiments and clinical studies.

NfL is a subunit of the neurofilament (NF) protein, which provides structural stability and maintains the integrity and pulse velocity of neurons. Some clinical studies found that patients with neurodegeneration have high levels of NfL in CSF; elevated levels of NfL in CSF and plasma could accurately distinguish healthy individuals from AD patients (Mattsson et al., 2017; Gaetani et al., 2019; Olsson et al., 2019). Modvig et al. (2016) reported that the NfL level in CSF predicts the visual outcomes after optic neuritis. In the event of degenerative changes in the retina of patients with nAMD, NfL will be released locally from the axons that degrade the retinal nerve fiber layer and spread to the vitreous fluid and AH, resulting in an increase in the concentration of NfL in AH, which was consistent with the findings of Subramanian et al. (2020). However, due to many diseases in the particular subject, the correlation between NfL levels and ophthalmopathy cannot be proved. Some other studies demonstrated that the decreased level of NfL in the ganglion cell layer of the retina and optic nerve is a response to induced injury or ischemia (Taniguchi et al., 2004; Sasaoka et al., 2006; Kamalden et al., 2011). The release of NfL in AH of cataract patients may be another mechanism. Therefore, the pathophysiological mechanism of AD biomarkers in AH needs to be explored further by neurologists, ophthalmologists, and basic researchers.

p-tau181 is also a known biomarker of neurodegeneration in CSF and blood (Hampel et al., 2018). Although both NfL and tau prote p-tau181 in AH ins are released from axons due to axonal degeneration, no correlation has been detected between NfL and, which is not consistent with the phenomenon in the brain and CSF. In addition, p-tau181 was not associated with Aβ in either nAMD or cataract patients’ AH, which might be due to the replacement of local sources of NfL and Aβ proteins in the eyes, such as the release of NfL from the retinal nerve fiber layer or amyloid plaques found in the retina independent of those found in the brain (Jin et al., 2019). The development of supersensitive immunoassay and new mass spectrometry technology has provided clinical data showing that p-tau181 can be used as a biomarker for early diagnosis of AD; however, additional studies are required to verify the diagnostic value of p-tau181 in AH.

Furthermore, lower MMSE scores indicated poor cognitive function, showing a negative correlation with the concentration of NfL in the AH of patients with nAMD. However, our data showed that the level of Aβ40, Aβ42, GFAP, or p-Tau181 was not associated with MMSE scores. In addition, the commercial spectral-domain OCTA was used to obtain the retina images of all patients, The correlation analysis between macular vessel density, retinal thickness, and AD biomarkers in the nAMD group explored the feasibility and effectiveness of retinal biomarkers measured by OCTA images as a diagnostic tool for early AD. However, OCTA images of the cataract group were not included in the analysis due to that phacoscotasmus caused poor retinal image quality and affected retinal stratification analysis. To the best of our knowledge, the association of AD biomarkers in AH and retinal specific markers of neurodegeneration obtained by OCTA or MMSE scores has not yet been elaborated. The current results lay a foundation for a large sample size cohort study in the future.

The highest advantage of this study is that the AH samples were obtained from patients with nAMD and cataracts. Unlike previous studies on the correlation between AD and ophthalmopathy, none of our subjects were diagnosed with any type of dementia, nor had any history of head injury or family history of cognitive disorders. This is helpful while exploring the potential of NfL, Aβ, and p-tau181 proteins as early diagnostic tools for AD using biological samples other than CSF and plasma. On the other hand, AH is a crucial component of the intraocular fluid and communicates with vitreous fluid. The expression of cytokines in AH reflects the physiological and pathological changes in lens and retina, while eyes and brain have a common embryological source and retina is the extension of cortical nerves; thus, intraocular fluid may be closer to the pathophysiological state of CSF than plasma and less affected by systemic diseases. Compared to the non-renewable vitreous fluid, AH is an ideal detection material. Because acquiring AH samples is more convenient and less traumatic to eyes, an appropriate amount can be obtained by anterior chamber puncture in the outpatient treatment or operating room. In this study, all AH samples of nAMD patients were collected by intravitreal injection treatment (Xing et al., 2014), and those from cataracts by phacoemulsification. These sampling procedures do not cause additional harm to the patient and can also prevent the increase in intraocular pressure caused by the injection of the drug into the eye (Saxena et al., 2019). Moreover, many eye diseases, such as nAMD and DR, were related to AD and routinely required multiple intravitreal injections (anti-VEGF treatment), facilitating multiple AH samples to observe the change rate of AD biomarkers.

Nevertheless, the present study has several limitations. Firstly, we could not obtain aqueous humor samples from healthy elderly because it is not in accordance with ethical conduct. Secondly, our sample size, limited by the scarcity of aqueous humor specimens, needs to be expanded to verify the correlation between AD biomarkers in AH and cognitive function in non-dementia patients. In addition, cataract patients had poor OCTA images due to lens opacity, which affected the accuracy of fundus retinal thickness and blood flow density measurement, so hence were not included in the statistical analysis. Finally, MMSE tests that are approximate assessments of cognitive function are less specific and effective than PET or MRI in detecting moderate dementia especially in mild dementia, and while it is highly specific and valid in detection of moderate dementia. Therefore, more sensitive way to assess cognitive function, including neurologist evaluation, detailed neuropsychological testing, and brain imaging would be needed to further verify the relationship between the AH biomarkers and development of dementia. Furthermore, future multicenter clinical studies with larger sample size and/or longitudinal study would be helpful to verify the correlation between AD biomarkers in AH and CSF and their correlation with brain imaging markers. Despite the above limitations, the current study provided an early AD diagnosis method that is less invasive, easy to obtain, less affected by systemic conditions, and can is widely applicable. Future directions include investigation to determine if tear secretions, which are easier to access and less invasive, contain biomarker levels that are associated with cognition, and a more comprehensive and sensitive way, including neurologist evaluation, detailed neuropsychological test, protein biomarkers in AH and OCTA images might be needed to further explore the clinical value of AD biomarkers in AH for early diagnosis of AD.

## Conclusion

NfL, Aβ40, Aβ42, GFAP, and p-tau181 can be detected in the aqueous humor and may play a role in early dementia detection in individuals at risk for AD. We observed that NfL, Aβ40, Aβ42, and p-tau181 levels in aqueous humor were influenced by the patients’ clinical eye conditions. Thus, this study served as a foundation for further investigation of the above AD-related biomarkers in ocular fluids. To the best of our knowledge, this is the first study that correlates neurocognition with AD-related proteins in the aqueous humor and macular microvascular parameters. The results suggested that the examination of AD-related biomarker content in human aqueous humor and OCTA may improve the ocular-based AD detection methods and forestall the progression of preclinical AD.

## Data availability statement

The original contributions presented in the study are included in the article/Supplementary material, further inquiries can be directed to the corresponding authors.

## Ethics statement

The studies involving human participants were reviewed and approved by the Medical Ethics Committee of The Shanghai Tenth People’s Hospital of Tongji University. All of the participants provided their written informed consent to participate in this study.

## Author contributions

JB participated in the data analysis and writing of the paper. ZW, MW, TW, YZ, and YX participated in data collection and revising the paper. QP and HX designed the study and wrote and revised the paper. All authors contributed to the article and approved the submitted version.

## Funding

This work was supported by the National Natural Science Foundation of China [grant number 81470025); the Shanghai Municipal Health Bureau [grant number ZY (2018–2020)-ZWB-1001-CPJS10); and the Three-Year Action Plan for Promoting Clinical Skills and Clinical Innovation in Municipal Hospitals (grant number SHDC2020CR5014).

## Conflict of interest

The authors declare that the research was conducted in the absence of any commercial or financial relationships that could be construed as a potential conflict of interest.

The reviewer NW declared a shared affiliation with the author HX to the handling editor at the time of review.

## Publisher’s note

All claims expressed in this article are solely those of the authors and do not necessarily represent those of their affiliated organizations, or those of the publisher, the editors and the reviewers. Any product that may be evaluated in this article, or claim that may be made by its manufacturer, is not guaranteed or endorsed by the publisher.
